# cGAS, an innate dsDNA sensor with multifaceted functions

**DOI:** 10.1016/j.cellin.2025.100249

**Published:** 2025-04-17

**Authors:** Yutong Liu, Pinglong Xu

**Affiliations:** a Department of Hepatobiliary and Pancreatic Surgery and Zhejiang Provincial Key Laboratory of Pancreatic Disease, The First Affiliated Hospital, School of Medicine, Zhejiang University, Hangzhou, 310058, Zhejiang, China; b Institute of Intelligent Medicine, ZJU-Hangzhou Global Scientific and Technological Innovation Center, Hangzhou, 310058, Zhejiang, China; c MOE Laboratory of Biosystems Homeostasis and Protection, Zhejiang Provincial Key Laboratory for Cancer Molecular Cell Biology, Life Sciences Institute, Zhejiang University, Hangzhou, 310058, Zhejiang, China; d Cancer Center, Zhejiang University, Hangzhou, 310058, Zhejiang, China

**Keywords:** cGAS-STING, Pattern recognition receptors, Innate immunity, Nucleic acid sensing, dsDNA, TBK1, IRF3, PERK, Autophagy, Interferon, Antitumor immunity, DNA damage, mtDNA, Organelle, Inflammation, Autoimmune diseases, Neurological diseases

## Abstract

Cyclic GMP-AMP synthase (cGAS) functions as a pivotal intracellular sensor for the innate immune sensing of double-stranded DNA (dsDNA), monitoring those nucleic acids from foreign and endogenous sources. Upon assembling into cellular condensates with dsDNA and regulators, cGAS synthesizes 2′3′-cGAMP that activates the downstream STING signaling. This activation triggers a variety of intracellular responses, including autophagy, mRNA translation, interferon signaling, and inflammatory responses. Context-dependently, cGAS resides in diverse cellular compartments, including the nucleus, micronuclei, plasma membrane, and organelle surfaces. Beyond its DNA-sensing role, cGAS can play complex roles in these locations, such as DNA damage repairing, membrane restoration, chromatin condensation, angiogenesis, and aging regulation. This comprehensive review summarizes recent advances in the activation, regulation, and pharmacological management of cGAS, focusing on its molecular mechanisms, post-translational modifications (PTMs), and therapeutic interventions. The functional implications of cGAS in various disease contexts, including infectious diseases, autoinflammatory diseases, autoimmune diseases, aging, and cancers, are also covered.

## 1 Overview of the cGAS-STING signaling mechanism

The innate immune systems employ versatile mechanisms to defend organisms against external pathogens and internal damage(Liu and Pu, 2023; Mantovani and Garlanda, 2023). When DNA is mislocalized, pattern recognition receptors (PRRs)(Kawai and Akira, 2010; Liu et al., 2016) detect this abnormality and trigger an arrange of immune and inflammatory responses(Dou et al., 2017; Zhang et al., 2020b). Toll-like receptor 9 (TLR9) was the first identified DNA sensor discovered by Akira et al. in 2000(Fitzgerald and Kagan, 2020; Hemmi et al., 2000). Since then, numerous DNA sensors with diverse functions have been documented, including absent in melanoma 2 (AIM2)(Bürckstümmer et al., 2009; Fernandes-Alnemri et al., 2009; Hornung et al., 2009; Roberts et al., 2009), DNA-dependent activator of interferon (IFN) regulatory factors (DAI)/Z-DNA binding protein 1 (ZBP1)(Takaoka et al., 2007), gamma-IFN-inducible protein 16 (IFI16)(Unterholzner et al., 2010), and inflammasomes(Muruve et al., 2008). Chen et al. identified cGAS (also called C6orf150 or MB21D1) as the primary cytosolic DNA sensor, highlighting its essential role in producing 2′3′-cyclic GMP-AMP (cGAMP), the second messenger for stimulator of interferon genes (STING, also called MITA, MPYS, ERIS or TMEM173) signaling(Sun et al., 2013). Consequently, collaborative efforts from several laboratories have established a novel innate immune cascade for sensing and responding to cytosolic dsDNA, known as the cGAS-STING signaling pathway(Ishikawa and Barber, 2008; Ishikawa et al., 2009; Sun et al., 2009, 2013; Wu et al., 2013; Zhong et al., 2008).

Under physiological conditions, cGAS activity is tightly regulated through autoinhibition. Upon binding aberrant self or foreign dsDNA in the cytoplasm, cGAS undergoes a conformational change that activates its catalytic site, leading to cGAMP production from GTP and ATP(Ablasser and Chen, 2019; Chen and Xu, 2023; Hopfner and Hornung, 2020; Kato et al., 2017; Wu et al., 2024c). The binding of cGAMP to the STING homodimer on the endoplasmic reticulum (ER) induces conformational changes and spatial translocation of STING proteins from the ER to the ER-Golgi intermediate compartment and the Golgi apparatus(Hopfner and Hornung, 2020). The C-terminal of STING recruits and activates TANK-binding kinase 1 (TBK1), leading to the phosphorylation of interferon regulatory factor 3 (IRF3)(Dobbs et al., 2015). Phosphorylated IRF3 dimerizes and translocates to the nucleus to induce type I IFNs and various IFN-stimulated genes (ISGs). This process has been extensively studied and defined as a canonical cGAS-STING signaling pathway. In addition, STING activation can also trigger the NF-κB signaling pathway that results mainly in the production of proinflammatory cytokines(Hubel et al., 2019; Li et al., 2013a; Zhang et al., 2019). However, whether this process depends on TBK1 remains a subject of debate.

## 2 Biological functions of cGAS-STING signaling

**Fig. 1:**
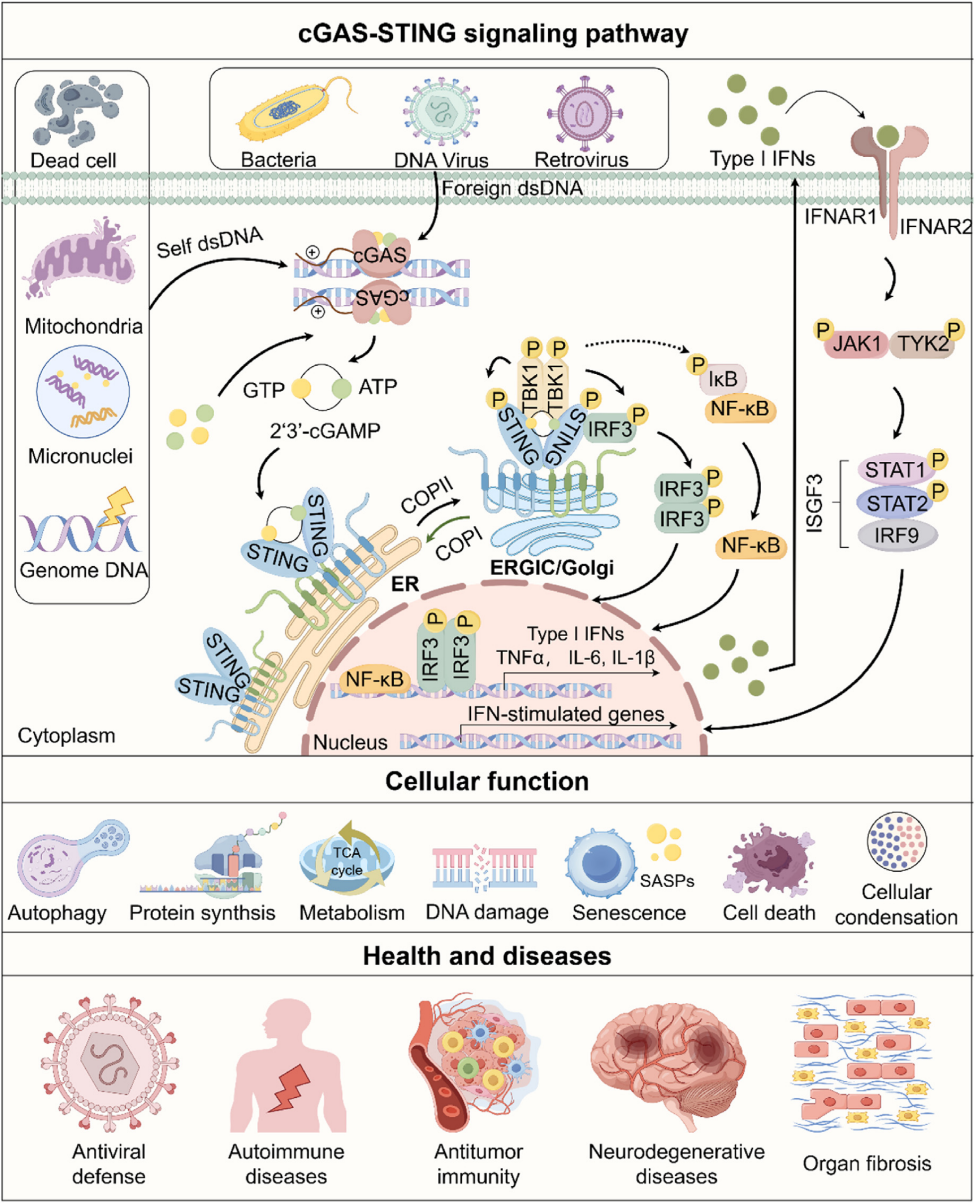
**The cGAS-STING pathway and its cellular and disease functions.** The cGAS-STING signaling pathway surveils cytoplasmic dsDNA to initiate innate immune responses. Free cGAS, a cytoplasmic PRR, lacks a functional active site in resting cells. In the presence of mislocalized dsDNA in the cytoplasm, either the pathogens or the host, cGAS would bind and induce a conformational change, facilitating the oligomerization that simultaneously sandwiches the two dsDNA strands and catalyzing the synthesis of the second messenger 2′3′-cGAMP. cGAMP binds to STING on the ER, inducing a 180° clockwise rotation of STING LBD and unraveling the right-handed crosslinked helices. Subsequently, STING translocates to the ERGIC and the Golgi apparatus, where it oligomerizes and recruits the signaling essential kinase TBK1 and the transcription factor IRF3 to form the canonical signalosome. Activated TBK1 further phosphorylates IRF3 and triggers its dimerization and nuclear translocation to induce the production of IFN-I and ISGs. Additionally, STING recruits IKKs to facilitate the transcription of proinflammatory cytokine genes through the NF-κB signaling pathway. cGAS-STING signaling plays a critical role in cellular functions and diseases through IFN-dependent and independent routes. **Abbreviations**: LBD, ligand-binding domain; ERGIC, ER-Golgi intermediate compartment; IFN-I, type I Interferon; IKKs, inhibitor of kappa B kinase; NF-κB, nuclear factor kappa-B; SASPs, senescence-associated secretory phenotypes. This figure was created using Figdraw.

As a dsDNA sensor, cGAS broadly recognizes dsDNA from both self- and foreign sources in a sequence-independent manner. Therefore, it serves as a crucial sentinel for tissue damage and infection. As has been demonstrated by numerous studies, the cGAS-STING pathway is involved in a wide range of cellular processes, including autophagy(Gui et al., 2019; Liu et al., 2018b; Moretti et al., 2017), protein synthesis(Franz et al., 2018; Wan et al., 2021; Zhang et al., 2022a), lipid and glucose metabolism(Hasan et al., 2017; Qiao et al., 2022; Vila et al., 2022; Zhang et al., 2022b; Zhao et al., 2018), condensates formation(Du and Chen, 2018; Liu et al., 2019; Meng et al., 2021; Zhou et al., 2021a), DNA damage repair(Banerjee et al., 2021; Jiang et al., 2019; Liu et al., 2018a), cellular senescence(Dou et al., 2017; Glück et al., 2017; Hou et al., 2021; Mackenzie et al., 2017; Wu et al., 2024b; Yang et al., 2017; Zhang et al., 2022a), and diverse types of cell death(Gulen et al., 2017; Li et al., 2021b; McArthur et al., 2018; Ning et al., 2019; Tang et al., 2016) through canonical and noncanonical pathways(Chen and Xu, 2023; Newman and Shadel, 2023; Zhang et al., 2020b). Moreover, at the systemic level, dysregulation of cGAS-STING signaling is tightly associated with a large subset of diseases, including Aicardi–Goutières syndrome (AGS)(Gao et al., 2015; Gray et al., 2015; Pokatayev et al., 2016), systemic lupus erythematosus (SLE)(An et al., 2017; Gao et al., 2015), STING-associated vasculopathy of infancy (SAVI)(Liu et al., 2014; Motwani et al., 2019), cancer(Bakhoum et al., 2018; Du and Chen, 2018; Jiang et al., 2019; Liu et al., 2018a; Meng et al., 2021; Yu et al., 2021; Zhou et al., 2024), fibrosis(Chung et al., 2019; Wu et al., 2024b; Zhang et al., 2022a), as well as neurodegenerative diseases including amyotrophic lateral sclerosis (ALS)/frontotemporal dementia (FTD)(McCauley et al., 2020; Yu et al., 2020), Parkinson's disease(Sliter et al., 2018), lysosomal storage diseases(Wang et al., 2024), glaucoma(Liu et al., 2024b) and Huntington's disease(Sharma et al., 2020) (Fig. 1). Recent research has increasingly focused on the specific functions of cGAS-STING in distinct cell types and tissue microenvironments. A comprehensive integration of these studies would deepen our understanding of the multifaceted roles of this signaling pathway across various environments and its specific activation mechanisms.

## 3 Mechanisms for dsDNA-induced activation of cGAS

### 3.1 Identification of cGAS and cGAMP

In 2013, Chen and colleagues identified cGAMP as an endogenous cyclic dinucleotide second messenger that directly binds to and activates STING, initiating downstream signaling cascades in mammals(Wu et al., 2013). Hornung, Patel, and Chen groups determined a noncanonical form of cGAMP, i.e., 2′3′-cGAMP, to mediate physiological STING signaling(Ablasser et al., 2013; Gao et al., 2013; Wu et al., 2013). Using quantitative mass spectrometry and conventional protein purification, Chen et al. established cGAS as the enzyme responsible for cGAMP synthesis(Sun et al., 2013). This seminal work spurred the identification of cGAS across diverse species, from fish to mammals, underscoring its evolutionary conservation. These investigations unveiled a novel and comprehensive innate immune signaling pathway: cGAS-cGAMP-STING-IRF3-IFN-I signaling. Subsequent research has extensively documented the capacity of cGAS to detect diverse dsDNA from self- or foreign sources, a process independent of DNA sequence. This broad DNA sensing capability naturally prompts the question: what is the structural basis for cGAS to recognize and bind DNA?

### 3.2 Structural basis for cGAS-mediated sensing of dsDNA

cGAS is primarily composed of a disordered and positively charged N-terminal domain, a C-terminal catalytic domain including an NTase core domain, a highly conserved Mab21 domain, and a newly identified dsDNA-binding domain (site C). Under normal physiological conditions, free cGAS lacks appropriate structural active sites. However, when aberrant DNA appears in the cytoplasm, crystallographic studies reveal that cGAS forms a 2:2 complex with dsDNA. During this binding process, cGAS undergoes a substantial conformational change that opens its active site and facilitates the formation of a dimer through surface hydrogen bonds. Each cGAS surface contains two DNA binding sites (sites A and B), which can simultaneously bind two dsDNA molecules in a sequence-independent manner. The two dsDNA molecules are stabilized and anchored between the cGAS dimer through electrostatic interactions and hydrogen bonds. Subsequently, the rearrangement of residues within the cGAS active site facilitates its activation. Furthermore, other residues within the zinc-ion-binding domain also play a crucial regulatory role in the cGAS dimerization(Civril et al., 2013; Zhang et al., 2020b). Interestingly, cGAS binds to DNA regardless of sequence but prefers DNA length and mechanical flexibility(Wang et al., 2022b). Studies have found that a 12 bp DNA sequence is ineffective in activating mouse cGAS, while sequences ranging from 16 to 18 bp demonstrate a high affinity *in vitro*. However, *in vivo* experiments indicate that approximately 45 bp of DNA is necessary to activate cGAS and subsequent downstream signaling effectively. It is worth noting that activation of human cGAS necessitates the presence of longer DNA fragments (>100 bp)(Li et al., 2013a; Zhou et al., 2018). This length dependency enhances the efficiency of responding to danger signals while minimizing background noise, which prevents interference from DNA fragments and low-concentration DNA, thereby improving the control over the immune surveillance function of organisms.

### 3.3 DNA conformation in cGAS binding

The affinity of cGAS for DNA is modulated by DNA conformation. B-DNA, characterized by its smooth, right-handed helical structure, represents the predominant form of DNA under physiological conditions. Structural analyses reveal that cGAS engages with the B-DNA backbone via its zinc-ion-binding domain(Civril et al., 2013). Z-DNA was initially considered a non-functional structural variant despite its limited genomic abundance. However, recent investigations have demonstrated that cGAS exhibits a markedly reduced binding affinity for Z-DNA compared to B-DNA. Furthermore, polyamines such as spermine and spermidine have been shown to promote the transition of DNA to the Z-DNA conformation, thereby effectively attenuating excessive cGAS activation(Zhao et al., 2023). This observation offers a novel approach to modulating cGAS-DNA interactions by maneuvering DNA conformation.

### 3.4 Phase separation in cGAS activation

**Fig. 2:**
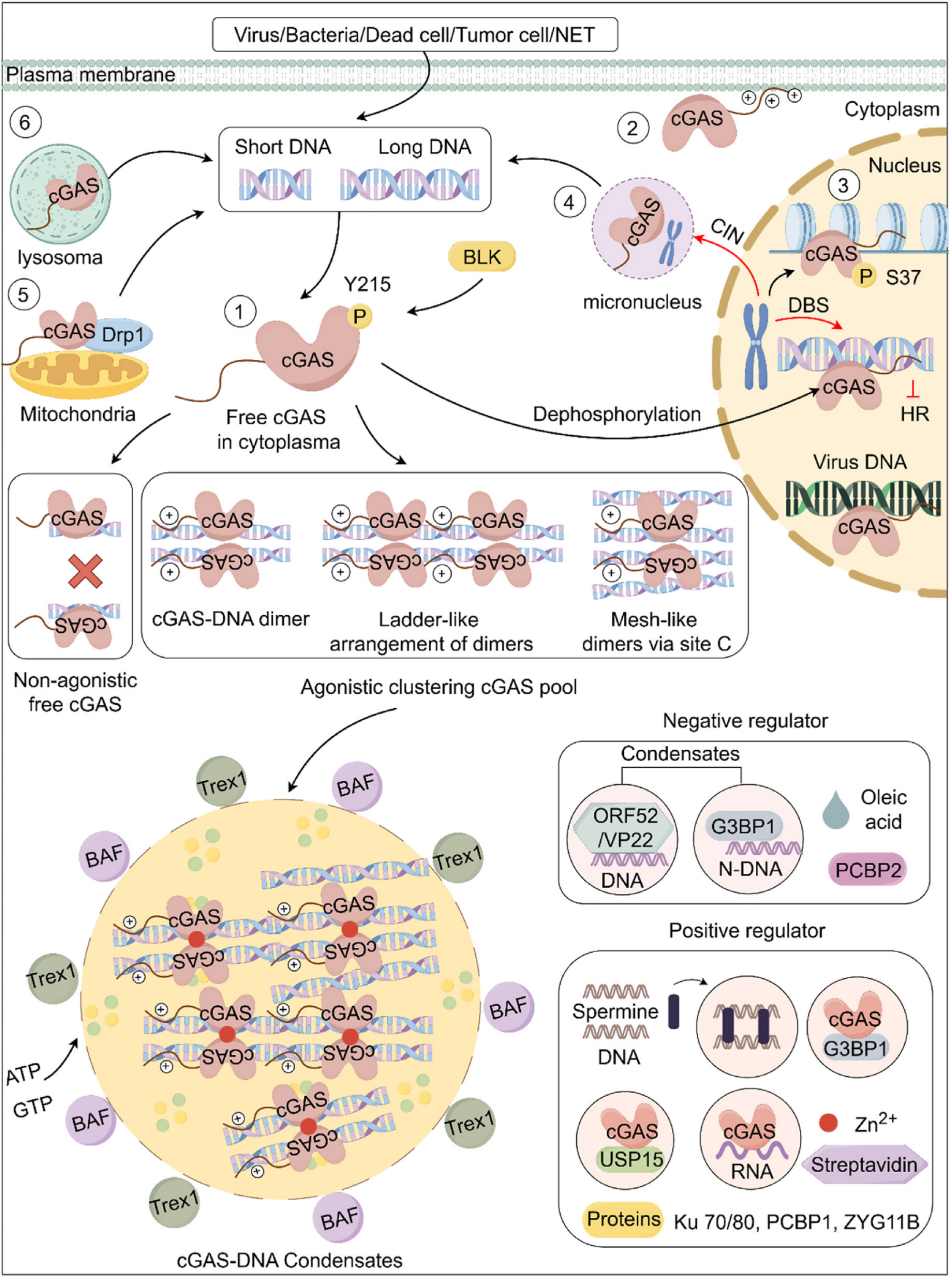
**Cellular Localization and Activation Mechanism of cGAS.** Beyond its established function as a cytosolic DNA sensor, cGAS exhibits a multifaceted subcellular distribution, localizing to the cytoplasmic membrane and various organelles, including the nucleus, micronuclei, mitochondria, and lysosomes (depicted in Fig. 2, panels 1–6). Under normal physiological conditions, the predominant fraction of cGAS remains inactive within the nucleus due to its interaction with chromatin. Upon activation by long DNA molecules originating from both exogenous and endogenous sources, cGAS initially assembles into a stoichiometric 2:2 complex with DNA. This initial complex then undergoes a series of dynamic rearrangements mediated by interactions involving N-terminal charged patches and DNA-binding site C domains within cGAS, resulting in more stable, higher-order structures resembling ladders and meshes. The progressive accumulation of these stable cGAS-DNA structures promotes liquid-liquid phase separation, forming cGAS-DNA condensates. Within this condensed, microreactor-like environment, cGAS achieves a high local concentration, which enhances the stability of cGAS dimerization and consequently amplifies its catalytic activity. Moreover, forming these condensates shields cGAS from negative influences exerted by TREX1 and BAF. **Abbreviations:** CIN, chromosomal instability.

In addition to length dependency, the binding affinity of cGAS to DNA exhibits concentration dependency. Chen et al. discovered that when cGAS and DNA are mixed *in vitro*, they form droplets with phase separation characteristics, which were subsequently defined as cGAS-DNA condensates. The droplets become more extensive as concentration increases, and cGAS and DNA exhibit dynamic behaviors. Notably, only full-length cGAS with long DNA tends to form larger droplets, consistent with previous findings(Du and Chen, 2018). Moreover, free Zn^2+^ ions can further facilitate phase separation, thereby enhancing the enzymatic activity of cGAS(Du and Chen, 2018). A new DNA binding site, site C, within the catalytic domain of cGAS, is crucial for forming cGAS-DNA condensates(Xie et al., 2019). These condensates function like miniature reactors, concentrating reactants, enzymes, ATP, and GTP. By precisely regulating the concentration, composition, and dynamics of molecules, it promotes the production of cGAMP and activation of downstream innate immune signaling in a more potent, independent, efficient, and precise manner. Moreover, DNA in this state can evade degradation by the three prime repair exonuclease (TREX1) through phase separation, ensuring sustained activation of cGAS. Additionally, access to barrier-to-autointegration factor (BAF) is restricted due to phase separation(Guey et al., 2020; Zhou et al., 2021b). In response to this crucial regulatory mechanism, organisms and pathogens have encoded various proteins that positively or negatively regulate the formation of cGAS condensates, achieving intricate functions (Fig. 2).

## 4 Cellular distribution and translocation of cGAS

Although initially identified as a cytoplasmic DNA sensor, the precise subcellular localization of cGAS is now recognized as essential for dsDNA sensing and subsequent activation of downstream signaling pathways. An intricate landscape of cGAS distribution has been documented, including those in mitochondria, lysosomes, plasma membranes, micronuclei, and the nucleus. The presence of cGAS in these diverse compartments appears to be tightly governed by poorly defined mechanisms. For example, Barnett et al. have shown that free cGAS exhibits binding to the plasma membrane via its N-terminal domain, thereby functioning as a membrane-localized protein to restrain its overactivation(Barnett et al., 2019). Wang and colleagues identified MYO1F-mediated recruitment of KAT2A, which acetylates cGAS and consequently facilitates its localization to the plasma membrane(Wang et al., 2025). The nuclear cGAS localization has been frequently observed(Zhao et al., 2020a), and a substantial proportion of cGAS is sequestered within the nucleolus, with its catalytic activity inhibited by the acidic patch of histone H2A-H2B heterodimers(Boyer et al., 2020; Michalski et al., 2020; Pathare et al., 2020). Kujirai et al. have reported that the three DNA-binding sites on cGAS are constitutively occupied when localized to the nucleus, effectively precluding its interaction with nuclear DNA(Kujirai et al., 2020). Upon viral infection or lysosomal dysfunction, cGAS undergoes evident translocation to the cytoplasm, where it executes canonical functions(Ablasser and Chen, 2019; Hopfner and Hornung, 2020; Wang et al., 2024). Intriguingly, cGAS can be enriched within micronuclei due to micronucleus membrane rupture(Bakhoum et al., 2018; Heijink et al., 2019; Joo et al., 2023; MacDonald et al., 2023; Mackenzie et al., 2017; Takaki et al., 2024). Adding more complexity, Qiu et al. reported the localization of cGAS to the outer mitochondrial membrane in tumor cells(Qiu et al., 2023), and lysosomal localization of cGAS has been suggested in the context of Huntington's disease and lysosomal storage diseases(Sharma et al., 2020; Wang et al., 2024). By advanced higher-resolution microscopy, live-cell imaging techniques, and cryo-electron tomography (cryo-ET), cGAS localization could be more precisely tracked. The complex regulation of cGAS translocation is unexpected and intriguing in cGAS-STING research.

## 5 PTMs for cGAS regulation

Highly conserved across diverse species, cGAS plays a pleiotropic role in some fundamental cellular processes(Dvorkin et al., 2024; Hopfner and Hornung, 2020; Skopelja-Gardner et al., 2022). Consequently, the precise and stringent regulation of cGAS activity and protein stability is paramount *in vivo*. Dysregulation of cGAS function is therefore conceivable as a trigger for aberrant innate immune activation, potentially contributing to the pathogenesis of a spectrum of diseases, including autoimmune disorders, neurodegenerative conditions, and organ fibrosis(Chen and Xu, 2023). PTMs are recognized as critical regulatory mechanisms in a multitude of biological processes, modulating protein transport, stability, intermolecular interactions, and functional activity(Yang et al., 2022a), and considered central to the intricate regulation of cGAS-STING-mediated innate immunity(Liu et al., 2013, 2016).

### 5.1 Phosphorylation

Phosphoinositide 3-kinase (PI3K)/AKT signaling is frequently observed to be hyperactive in cancer. Intriguingly, AKT-mediated phosphorylation of cGAS at serine 291 (Ser291) has inhibited its enzymatic activity and attenuated the immune response to viral infection(Seo et al., 2015). During mitosis, the cyclin-dependent kinase 1 (CDK1)-cyclin B kinase complex can also phosphorylate human cGAS, resulting in a diminished capacity for cGAMP synthesis and preventing aberrant cGAS activation during cell division(Zhong et al., 2020). Furthermore, Bruton's tyrosine kinase (BLK)-mediated phosphorylation of human cGAS at Tyr215 maintains its cytoplasmic localization(Liu et al., 2018a), while dephosphorylation at Tyr215 occurs upon detection of aberrant free dsDNA, which activates downstream signaling. DNA-dependent protein kinase (DNA-PK) has also been shown to phosphorylate cGAS at T68 or S213, inhibiting cGAS enzymatic activity(Sun et al., 2020). The mechanism target of rapamycin complex 2 (mTORC2) also phosphorylates cGAS at S37, facilitating its localization to chromatin(Lv et al., 2024). These chromatin-bound cGAS (ccGAS) subsequently regulate cell growth and acquired drug resistance through epigenetic patterning, independent of STING signaling. Intriguingly, spleen tyrosine kinase (SYK) is activated upon endocytosis of viruses and phosphorylates cGAS, initiating downstream innate immune response(Yang et al., 2022b). It is evident that the delicate equilibrium between phosphorylation and dephosphorylation of cGAS, orchestrated by a diverse array of kinases and phosphatases, is crucial for maintaining cellular homeostasis and immune response.

### 5.2 Ubiquitination

cGAS undergoes K48-linked ubiquitination in quiescent cells at K414, initiating p62-mediated degradation via the proteasomal or autophagic-lysosomal pathways(Chen et al., 2016a). A recent investigation has revealed that nuclear cGAS degradation is also governed by the ubiquitin-proteasome system (UPS), wherein SPSB3 ubiquitinates nuclear cGAS through interaction with the cullin-RING ubiquitin ligase 5 (CRL5) complex(Xu et al., 2024). MARCH8 can also catalyze lysine-63 (K63)-linked ubiquitination of cGAS at K411, which disrupts its DNA binding capacity and consequently attenuates cGAMP production(Yang et al., 2022a). Listerin promotes K63-linked ubiquitination of cGAS by recruiting the E3 ubiquitin ligase TRIM27, which subsequently facilitates cGAS trafficking to endosomes for degradation(Qin et al., 2023). These findings propose the distinct roles of ubiquitin chain linkages in regulating diverse cellular functions of cGAS. Conversely, deubiquitinating enzymes (DUBs) modulate cGAS stability through the mediation of its deubiquitination(Zhang et al., 2020a). USP29, USP27X, and USP14 deubiquitinate K48-linked polyubiquitin chains on cGAS at K271 during DNA virus infection, which stabilizes cGAS proteins and potentiates its antiviral immune response(Chen et al., 2016a; Guo et al., 2019; Zhang et al., 2020a). Additionally, the deubiquitinase OTUD3 directly binds dsDNA and is recruited to cytoplasmic DNA complexes, from which it physically interacts with cGAS. This interaction stabilizes cGAS and enhances its enzymatic activity, promoting antiviral immunity against DNA viruses(Cai et al., 2022; Lyu et al., 2023). This reversible ubiquitination and deubiquitination collectively serve as crucial means to control the stability and activity of cGAS.

### 5.3 SUMOylation

Recent investigations have elucidated that cGAS is also subject to regulation by SUMOylation, both in quiescent cellular states and in the context of viral infection. Given that certain members of the TRIM family can function as E3 SUMO ligases, their potential to catalyze cGAS SUMOylation has been recently explored. TRIM38 can catalyze SUMOylation of cGAS at K217 in the resting cells to attenuate cGAS degradation(Hu et al., 2016). Infection of HSV-1 also leads to the SUMOylation of cGAS at K464, which enhances cGAS stability. SUMOylation at K335, K372, and K382 was also reported during HSV-1 infection that attenuated its DNA-binding capability, although specific E3 ligases catalyzing these modifications remained to be identified(Cui et al., 2017). On the contrary, sentrin-specific protease 7 (SENP7) promotes deSUMOylation of these lysine residues, potentiating cGAS activation(Cui et al., 2017). These findings suggest SUMOylation is another important mode of regulating this dsDNA sensor, though the intricate relations among ubiquitination, acetylation, methylation, and sumoylation need to be clarified.

### 5.4 Acetylation

Acetylation, a pivotal post-translational modification, plays a regulatory role in diverse biological processes to modulate protein function, chromatin architecture, gene expression, cell cycle progression, mitochondrial biology, and nucleocytoplasmic transport(Choudhary et al., 2009; Shvedunova and Akhtar, 2022). In quiescent cells, lysine residues, specifically K384, K394, and K414, located within the C-terminus of cGAS, undergo acetylation to attenuate cGAS enzymatic activity. Consistent with this observation, aspirin can directly acetylate K384 and K414, effectively inhibiting cGAS-mediated immune responses(Dai et al., 2019; Elkon, 2019; Lee et al., 2022). Conversely, HDAC3-mediated deacetylation of these C-terminal lysine residues is triggered upon aberrant cytoplasmic dsDNA accumulation, thereby relieving this acetylation-dependent inhibition of cGAS and potentiating the signaling(Dai et al., 2019; Song et al., 2020a). Intriguingly, acetyl-mimetic mutations at lysine residues K384 and K414 have been shown to diminish the capacity of cGAS to induce cell apoptosis, whereas a mutation at K198 enhances cGAS-dependent IFN signaling(Dai et al., 2019). Despite these insights, the specific acetyltransferases responsible for acetylation events remain to be identified. On the other hand, KAT5 can catalyze acetylation at multiple lysine residues within the N-terminus of cGAS (K47, K56, K62, and K83), thereby augmenting its DNA binding affinity and promoting innate immune response(Song et al., 2020b). The divergent functional roles of acetylation modifications within distinct domains of cGAS are particularly noteworthy, suggesting that distinct combinations of acetylation at specific lysine residues may orchestrate diverse regulatory mechanisms governing cGAS function.

### 5.5 Methylation

Ma et al. found that the protein arginine methyltransferase 5 (PRMT5) directly interacts with cGAS and catalyzes symmetric dimethylation of R124 in quiescent cells, which impairs the DNA-binding affinity of cGAS and thereby precludes aberrant cGAS activation(Ma et al., 2021). Liu and colleagues identified two additional members of the PRMT family, PRMT1(Liu et al., 2023b, 2024a) and PRMT3(Zhu et al., 2023), capable of methylating cGAS at R133 and R111, respectively, and these events inhibit cGAS dimerization and, consequently, cGAS-STING activation. PRMT1 has been implicated in enhancing immune evasion, suggesting that investigations into cGAS methylation may provide novel insights into the mechanisms of cancer immunology. Consistent with this notion, PRMT1 inhibitors exhibit synergistic effects with immune checkpoint blockade in enhancing cancer immunity and are currently under evaluation in phase I clinical trials(Liu et al., 2023b). Beyond arginine methylation, lysine residues on cGAS are also subject to methylation. Fang et al. discovered that SUV39H1, a lysine methyltransferase (KMT), catalyzes cGAS methylation at K362, promoting chromatin retention and attenuating cGAS activity. Intriguingly, serine deprivation impedes this methylation event and can facilitate cGAS release from chromatin and subsequent reactivation(Fang et al., 2023). Therefore, dietary serine restriction has emerged as an intervening strategy with the potential for cancer treatment. These findings suggest that methyltransferases may represent promising therapeutic targets for modulating cancer immunotherapy through cGAS-STING.

### 5.6 Palmitoylation

Palmitoylation of STING at the Golgi apparatus has been established as a critical modification for the subsequent activation of downstream innate immune responses(Mukai et al., 2016). Analogously, cGAS, in a non-quiescent state, also undergoes palmitoylation, independent of cGAMP or STING signaling(Shi et al., 2022). While palmitoylation typically modulates the function of membrane-associated proteins by influencing their subcellular localization or inducing conformational changes, it is noteworthy that this modification, in the case of cGAS, does not appear to exert its regulatory effects through localization alterations(Shi et al., 2022). Shi et al. identified C474 as the primary palmitoylation site on cGAS and determined that ZDHHC18 is the responsible cognate acyltransferase. This palmitoylation attenuates cGAS binding to dsDNA and impedes cGAS dimerization and subsequent activation(Shi et al., 2022). Conversely, palmitoylation at C404/405, mediated by ZDHHC9, paradoxically enhances cGAS enzymatic activity(Fan et al., 2023). Lysophospholipase-like 1 (LYPLAL1) has been identified as an enzyme that induces depalmitoylation of cGAS, resulting in impaired cGAS enzymatic activity. Therefore, pharmacological inhibition of LYPLAL1 significantly potentiates cGAS-mediated immune responses(Fan et al., 2023). This seemingly contradictory finding suggests that when catalyzed by distinct acyltransferases at different sites, palmitoylation may induce subtle alterations in the structural characteristics of cGAS and elicit disparate functional consequences.

### 5.7 Other modifications: glutamylation, NEDDylation, PARylation, and lactylation

Beyond the well-characterized PTMs previously discussed, recent investigations have identified additional regulatory modifications of cGAS. Glutamylation, a reversible PTM catalyzed by the tubulin tyrosine ligase-like (TTLL) family enzymes, occurs at E272 and E302 and attenuates either catalytic activity or the DNA-binding capacity of cGAS(Xia et al., 2016). Conversely, carboxypeptidase glutamate (CCP) enzymes catalyze deglutamylation at both sites to reactivate cGAS. Xiong and colleagues discovered that ARIH1, an E3 ubiquitin ligase, directly interacts with cGAS and unexpectedly catalyzes ISG15 monoubiquitination at K187, which promotes cGAS oligomerization and potentiates antiviral immunity against HSV-1 infection(Xiong et al., 2022). Li and colleagues identified RNF111 as a specific NEDD8 E3 ligase for cGAS, mediating NEDDylation at K231 and K421, promoting cGAS dimerization and dsDNA binding(Li et al., 2021a). Intriguingly, both RNF111 and RNA polymerase II subunit A C-terminal domain phosphatase-like 1 (RNA168) are implicated in cGAS NEDDylation and DNA damage repair processes, balancing DNA repair and damage-induced immune responses. Additionally, Wang et al. reported cGAS PARylation. Upon viral infection, DNA damage triggers the translocation of nuclear poly(ADP-ribose) polymerase 1 (PARP1) to the cytoplasm, interacts with cGAS, and catalyzes PARylation at D191, thus blocking dsDNA binding(Wang et al., 2022a). Recent research suggests that cGAS can undergo lactylation to lose its phase separation potentiality through a modification mediated by alanine-tRNA synthetases (AARS1 and AARS2) (Li et al., 2024), or promotes its ubiquitin-independent degradation(Rao et al., 2025), or conversely, stabilizes cGAS for stronger IFN-I responses(Zhang et al., 2024). Therefore, the modification residues and effects of cGAS lactylation could be context and scenario-dependent.

**Fig. 3:**
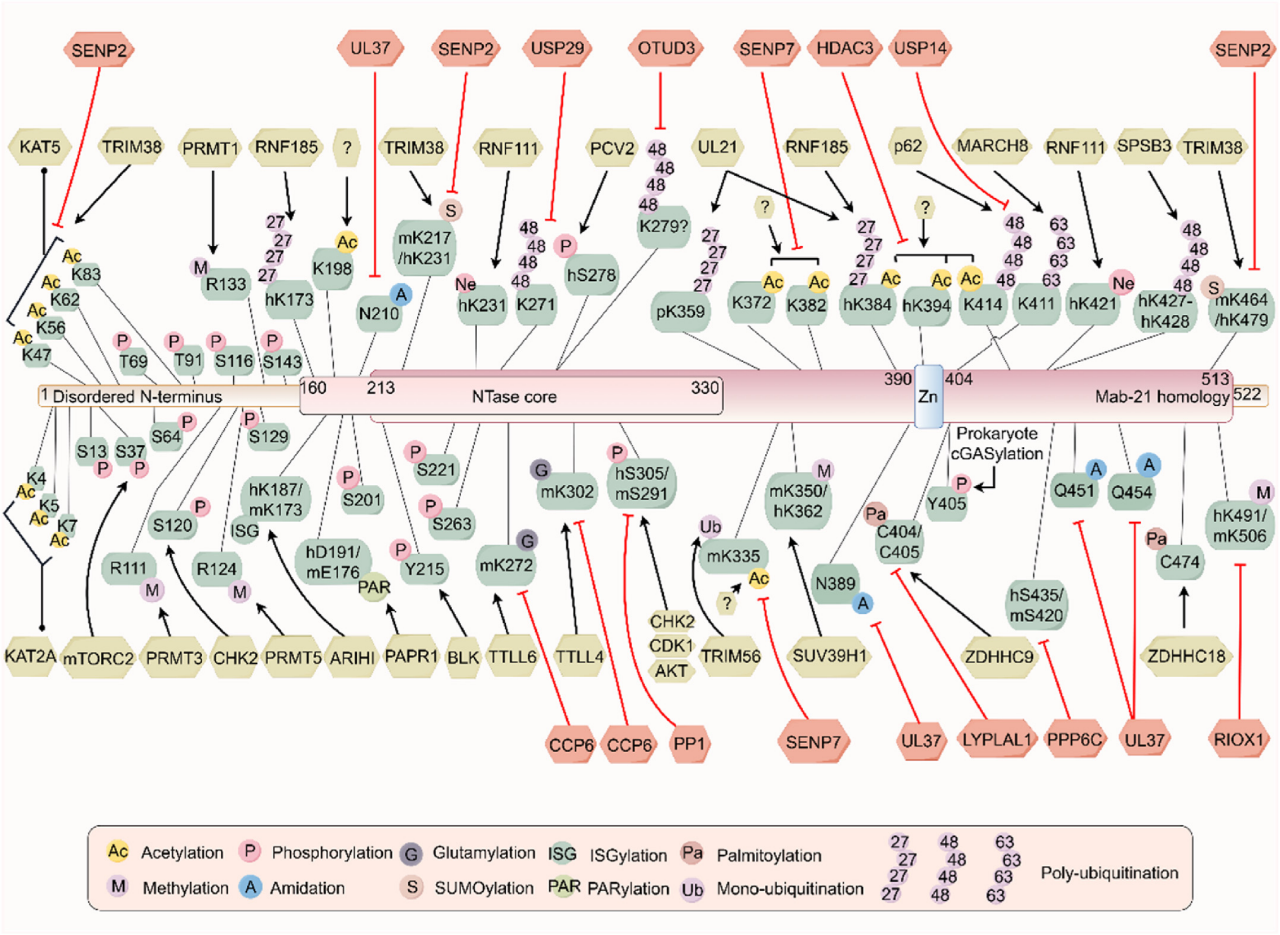
**The domains and PTMs of cGAS.** Human cGAS, a protein of 522 amino acids exhibiting significant structural conservation across species, comprises three distinct domains: a disordered N-terminal domain, an NTase core domain, and a Mab21 domain. Analogous to the N-terminal domain, the Mab21 domain harbors a highly conserved zinc-ion-binding domain essential for maintaining the tertiary structure and ensuring normal physiological function, serving as the region for dsDNA engaging. Under physiological and pathological conditions, cGAS undergoes dynamic regulation by a diverse array of PTMs, including ubiquitination, acetylation, glutamylation, phosphorylation, palmitoylation, methylation, amidation, SUMOylation, ISGylation, and PARylation, which enable precise and fine-tuned controls of both stability and enzymatic activity of cGAS. A schematic diagram illustrating the PTM sites on cGAS and the corresponding modifying enzymes is provided in Fig. 3. For a more comprehensive overview of their functions and further details, please refer to Table 1, Table 2

In summary, cGAS activity is intricately regulated by a complex interplay of PTMs that finely tune its function in cellular immune responses (Fig. 3) (Table 1, Table 2). Besides identifying novel PTM sites and functions of cGAS, exploring the intricate interactions among distinct cGAS PTMs in the context of physiological and pathological scenarios is intriguing. It is crucial to investigate whether distinct combinations of modifications might exhibit synergistic or antagonistic effects, which could afford more precise regulation of cGAS function and stability. Moreover, a more in-depth understanding of the spatial and temporal regulation of cGAS modification states under diverse pathological conditions is warranted.

**Table 1:** PTMs of cGAS with functional descriptions.

PTMs	Site	Mediator	Function	Refs
**Ubiquitination**	hK411/mK410	MARCH8	Inhibits the DNA binding ability and enzymatic activity of cGAS	Yang et al. (2022a)
	hK173, hK384	RNF185	Promotes enzymatic activity of cGAS	Wang et al. (2017a)
	hK427-428	SPSB3	Keep cGAS in check and ubiquitination degrades nuclear cGAS	(Chen, 2024; Xu et al., 2024)
	mK335	TRIM56	Promotes the dimerization and DNA binding ability of cGAS	Seo et al. (2018)
	Unknown	TRAF6	Promotes cGAS activation	Chen and Chen (2019)
	Unknown	RINCK		Liu et al. (2018c)
	Unknown	HBx	Promotes cGAS degradation	Chen et al. (2022)
	hK414	p62		Chen et al. (2016a)
	hK384/pK359	UL21		Ma et al. (2023)
	Unknown	Listerin-TRIM27		Qin et al. (2023)
	Unknown	HSP27		Li et al. (2022b)
**cGASylation(like Ub)**	Y405 (Prokaryote)	E2	Promotes cGAS activation and increases the affinity of cGAS to ATP	Yan et al. (2024)
**Deuniquitination**	hK414	TRIM14, USP14	Improves cGAS stability	Chen et al. (2016a)
	Unknown	UAF1-USP		Yu et al. (2024)
	Unknown	USP27X		Guo et al. (2019)
	mK271	USP29		Zhang et al. (2020a)
	hK279/mK265	OTUD3	Promotes the dimerization and DNA binding ability of cGAS	(Cai et al., 2022; Lyu et al., 2023)
**Acetylation**	hK47, hK56, hK62, hK83	KAT5	Promotes DNA-binding ability and activation of cGAS	Song et al. (2020b)
	K4, K5, K7	KAT2A	May have multiple roles in the nucleus and cytoplasm to regulate cGAS	Tang et al. (2021)
	K131, K292,K421	KAT2A	Regulates the membrane localization of cGAS	Wang et al. (2025)
	hK384/hK394/hK414	Unknown	Inhibits cGAS-dependent apoptosis	Dai et al. (2019)
	hK198	Unknown	Increases cGAS-mediated Cytokine Induction	Dai et al. (2019)
	Unknown	Aspirin	Inhibits cGAS-induced immune responses and self-DNA-induced autoimmunity	Dai et al. (2019)
**Deacetylation**	hK384/hK394/hK414	HDAC3	Promotes cGAS activation	Liao et al. (2020)
**Palmitoylation**	hC474	ZDHHC18	Inhibits enzymatic activity of cGAS and reduces cGAS dimerization and inhibits DNA bing to cGAS	Shi et al. (2022)
	hC404/hC405	ZDHHC9	Promotes the dimerization, DNA-binding ability, enzymatic activity and activation of cGAS	Fan et al. (2023)
	Unknown	QP383R	Inhibits the DNA binding ability, dimerization and enzymatic activity of cGAS	Hao et al. (2023)
**Depalmitoylation**	hC404/hC405	LYPLAL1	Inhibits the activation of cGAS	Fan et al. (2023)
**Sumoylation**	mK217/hK231, mK464/hk479, hk83	TRIM38	Improves cGAS stability	Hu et al. (2016)
	hK347/mK335, hK384/mK372, hK394/mK382	Unknown	Inhibits the DNA-binding, oligomerization and nucleotidyl-transferase activities of cGAS	Cui et al. (2017)
**Desumoylation**	mK217/hK231, mK464/hk479, hk83	SENP2	Promots cGAS degradation	Hu et al. (2016)
	hK347/mK335, hK384/mK372, hK394/mK382	SENP7	Promotes cGAS activation	Cui et al. (2017)
**ISGylation**	hK187/mK17	ARIHI	Promotes oligomerization level of cGAS	Xiong et al. (2022)
**PARylation**	hD191/mE176	PARP1	Inhibits the DNA binding ability of cGAS	Wang et al. (2022a)
**Methylation**	hR124	PRMT5	Inhibits the DNA binding ability of cGAS	Ma et al. (2021)
	hR133	PRMT1	Inhibits cGAS dimerization	Liu et al. (2023b)
	hK362, mK350	SUV39H1	Inhibits cGAS activity	Fang et al. (2023)
	hR111	PRMT3	Inhibits the DNA-binding ability and oligomerization of cGAS	Zhu et al. (2023)
**Demethylation**	mK491/hK506	RIOX1	Promotes cGAS binding to PAR and impedes HR repair	Xiao et al. (2022)
**Glutamylation**	hK286/mK272	TTLL6	Inhibits the DNA binding ability of cGAS	Xia et al. (2016)
	hK314/mK302	TTLL4	Inhibits the activity of cGAS	Xia et al. (2016)
**Deglutamylation**	hK286/mK272	CCP6	Promotes the DNA binding ability of cGAS	Xia et al. (2016)
	hK314/mK302	CCP5	Promotes the activity of cGAS	Xia et al. (2016)
**Phosphorylation**	hY215	BLK	Enhances the cytosolic retention of cGAS	(Jiang et al., 2019; Liu et al., 2018a)
	hT68, hS213	DNA-PK	Inhibits the activity of cGAS	Sun et al. (2020)
	mS291/hS305	AKT/CDK1		(Seo et al., 2015; Zhong et al., 2020)
	hS278	PCV2		Wang et al. (2021)
	hS120, hS305	CHK2	Inhibits cGAS-dependent Apoptosis	Zhen et al. (2023)
	Unknown	SRC	Inhibits enzymatic activity and DNA binding activity of cGAS	Dunker et al. (2023)
	S13, S37, S64, T69, T91, S116, S129, S143	AurB and other kinases	Blocks cGAS liquid phase separation and activation	Li et al. (2021c)
	S435	Unknown	Activates the nucleotidyltransferase activity	Li and Shu (2020)
	S37	mTORC2	Promoting the chromatin location of cGAS	Lv et al., 2024
	hY214/mY200, hY215/mY201	SYK	Promotes cGAS activation	Yang et al. (2022b)
**Dephosphorylation**	hS435/mS420	PPP6C	Inhibits the activity of cGAS	Li and Shu (2020)
	mS291/hS305	PP1	Restores the activity of cGAS	Zhong et al. (2020)
**Neddylation**	hK231, hK421	RNF111	Promotes dimerization and the DNA-binding ability of cGAS	Li et al. (2021a)
**Lactylation**	hK131/mK156	AARS1/2	Inhibits the activity of cGAS	(Li et al., 2024, Rao et al., 2025, Zhang et al., 2024)
**Deamication**	hN210	UL37	Reduces cGAS-dependent cGAMP production and innate immune signaling induced by dsDNA	Zhang et al. (2018)
**Cleavage**	D140, D157	Caspase 1	Inhibits IFN-I signaling	Wang et al. (2017b)
	D319	Caspase 3		(Ning et al., 2019; Ning and Wang, 2019)

h: human; m: mouse; p: pig.

**Table 2:** Interacting binding partners of cGAS.

Protein	Function	Interacting domain	Interacting domain of cGAS
PQBP1	Specifically enables sensing of HIV-1 cDNA by cGAS	The N-terminal fragment containing the WW domain and additional C-terminal regions	Unknown
G3BP1	Facilitates DNA-cGAS interaction and higher-order complex formation	The full-length protein	The N-terminal domain
ZCCHC3	Forms a complex with cGAS and increases affinity for DNA	The full-length protein	The NTase fold (213–382 aa) and the C-terminal fragment (383–522 aa)
Beclin-1	Inhibits cGAS activation	The central CCD (1–2)	The central NTase domain
PCBP1	Enhances DNA binding of cGAS	The KH domains	The C-terminal fragment (161–522 aa)
OASL	Negative regulator of cGAS, decreases cGAMP output	OASL-ΔUBL	Unknown
PCBP2	Decreases cGAS enzyme activity by antagonizing cGAS condensation	The KH3 domain	The C-terminal domain
Caspase-1	Cleaves cGAS and inhibits cGAS activation	p20 fragment of caspase-1	The N-terminal (D140 and D157)
KU70/80	Facilitates DNA-cGAS interaction and promotes enzymatic activity of cGAS	A central Ku core domain (Core) of KU 80	C-terminal catalytic domain
Rnf111	Promoted the dimerization and enhanced the DNA-binding ability of cGAS	Multiple binding domains of RNF111 (independent of the sumo-interacting motif (SIM) and RING domain)	The regions of 1–120 aa and 241–380 aa
p62	Mediated Selective Autophagic Degradation of cGAS	The UBA domain	C terminus (K414)
RNF185	Promotes enzymatic activity of cGAS	The RING domain (amino acids 39–80)	The C-terminal domain (201–522 aa)
TRIM56	Promotes the dimerization and DNA binding ability of cGAS	The C-terminal NCL1-HT2A-Lin41 (NHL) homologous region	The N-terminal RD
TRIM14	Inhibits autophagic degradation of cGAS and stabilizes cGAS	The PRYSPRY domain of TRIM14	C terminus of cGAS
MARCH8	Inhibites the DNA binding ability of cGAS	Zinc finger and transmembrane domains (TMDs)	The C-terminal region
TRAF6	Facilitates cGAS activation	The TRAF domain (348aa-522aa)	Unknown
E2	Facilitates cGAS activation and increased the affinity of cGAS to ATP	Unknown	The C-terminal region
UL21	Promotes cGAS degradation in the lysosome	The N-terminal region (1–200 aa)	The internal domain (290–400 aa)
SPSB3	Keep cGAS in check and ubiquitination degrades nuclear cGAS	Five variable loops extending from the bent β-sandwich core	The C-terminal helix (497–515 aa)
USP29	Improves cGAS stability	The ubiquitin carboxyl-terminal hydrolase (UCH) domain	The C-terminal domain containing the NTase domain
OTUD3	Improves cGAS stability and promotes enzymatic activity of cGAS	The OTU domain (1–183 aa)	The N-terminal domain (NTD) or NTase
ZDHHC18	Inhibits enzymatic activity of cGAS and reduces cGAS dimerization and inhibits DNA bing to cGAS	The nonmembrane region consisting of the N- and C-terminal portions	The C-terminal domain
QP383R	Inhibits the DNA binding ability, dimerization and enzymatic activity of cGAS	C-terminal tail (284–383 aa)	Both enzymatically active core (135–305 aa) and the deletion of RD domain (135–495 aa)
SENP7	Promotes the activity of cGAS	The N-terminal domain (1–300 aa)	The middle region (240–380 aa)
ARIH1	Promotes oligomerization level of cGAS	Multiple domains except for the N-terminal UBA domain	The C-terminal domain (151–552 aa)
TTLL4	Inhibits the activity of cGAS	Unknown	A fragment consisting of residues 280–320
TTLL6	Inhibits the DNA binding ability	Unknown	A fragment consisting of residues 240–280
PRMT5	Inhibits the DNA binding ability of cGAS	Methyltransferase (MTase) domain (308–637 aa)	The N-terminal domain (1–160 aa)
LC3	Promotes the autophagy of micronuclei	Unknown	LIR domain
H2AX	Promotes the recruitment of cGAS to DNA-damage sites	The phospho-epitope (pS139)	The C-terminal domain
NONO	Associates with cGAS in the nucleus and facilitates sensing of HIV DNA	Unknown	Unknown
LAMTOR1	Mediates DNA Fragments-Induced cGAS Degradation	The W102, Y140, Q145, and R147 residues	The L174, R339, and Q473 residues

## 6 Deregulation of cGAS in diseases

### 6.1 Infectious diseases

The cGAS-STING pathway is now firmly established as a critical component of the innate immune system, serving as a primary defense mechanism against foreign pathogens(Zhang et al., 2020b). Viral infections, such as HSV-1, can induce mitochondrial stress and the subsequent leakage of mitochondrial DNA (mtDNA) into the cytoplasm(Li et al., 2013b; West et al., 2015). Consequently, besides directly surveilling viral DNA in the nucleus(Lahaye et al., 2018), cGAS also detects these aberrant mtDNA byproducts to trigger an innate immune response. In retroviruses, such as HIV-1, cGAS is recruited to the HIV-1 capsid through direct interaction with PQBP1, thereby initiating antiviral responses(Herzner et al., 2015; Yoh et al., 2015, 2022). Beyond viral pathogens, bacteria represent another class of common infectious agents capable of activating cGAS-STING signaling. During infections with *Pseudomonas aeruginosa* and *Mycobacterium tuberculosis*, cGAS senses bacterial genomic DNA to initiate selective autophagy, leading to bacterial clearance(Watson et al., 2015). mtDNA is released during *Salmonella enterica serovar Typhimurium* infection to induce cGAS activation. During distinct phases of *Burkholderia pseudomallei*(Ahn and Barber, 2019; Hahn et al., 2018) or *Burkholderia thailandensis* infection, cGAS-STING employs divergent mechanisms to inhibit bacterial over-replication(Ku et al., 2020; Place et al., 2020). Upon infection with the parasite *Plasmodium*(Souza et al., 2020; Wang et al., 2019b) and *Toxoplasma*(Sun and Cheng, 2020; Wang et al., 2019b), cGAS senses parasitic genomic DNA and restricts parasite proliferation by activating signaling cascades and inducing IFN-Is. However, the precise role of cGAS-STING signaling in resisting pathogens could be complex and context-dependent. Mice deficient in cGAS-STING signaling exhibit paradoxical increases in resistance to *Schistosoma mansoni*(Souza et al., 2020) and the lethal *Plasmodium yoelii* YM strain(Yu et al., 2016), highlighting the diverse nature of this pathway in host-pathogen interactions.

### 6.2 Autoimmune and autoinflammatory diseases

The sequence-independent nature of dsDNA-cGAS interaction allows cGAS to recognize both foreign and self-DNA. Overactivation of cGAS significantly contributes to the development of both autoimmune and autoinflammatory diseases, including rheumatoid arthritis (RA)(Wang et al., 2019a), AGS(Gao et al., 2015; Gray et al., 2015; Pokatayev et al., 2016), and SLE(An et al., 2017; Gao et al., 2015; Kato et al., 2018). In patients with RA, dsDNA and cGAS expression upregulation has been observed, and cGAS deficiency in RA mice has been shown to exhibit a mitigated phenotype(Wang et al., 2019a). AGS(Gao et al., 2015; Gray et al., 2015; Pokatayev et al., 2016) is an autoimmune disease caused by single-gene mutations in proteins involved in DNA degradation, repair, and packaging, including TREX1, SAMHD1, RNaseH2, and LSM11. Dysregulated cytoplasmic DNA metabolism by these mutations overactivates cGAS-STING signaling. Similarly, elevated cGAS expression is seen in the serum of SLE patients(An et al., 2017; Kato et al., 2018), triggering significant IFN-Is, though the involvement of cGAS in SLE is complex and depends on disease severity and stage. During Myotonic dystrophy type 2 (DM2) development, nucleotide sequence (CCTG) repeat expansions lead to chronic endoplasmic reticulum stress accompanied by mtDNA leakage, subsequently activating cGAS(Rösing et al., 2024).

### 6.3 Cancers

The cGAS–STING pathway is multifaceted and influences tumor initiation and progression. Tumor cells frequently harbor genomic instability, sustain DNA damage, and endure cell cycle dysregulation and mitochondrial stress, accumulating free cytosolic dsDNA to trigger cGAS activation(Crasta et al., 2012; Riley et al., 2018), thus initiating a potent IFN-dependent innate immune response and recruiting cytotoxic effectors to mount antitumor immunity(Gao et al., 2013; Khoo and Chen, 2018; Lam et al., 2014; Marcus et al., 2018), mainly through helper T lymphocytes and natural killer cells. Besides, cGAS activation in tumor cells induces senescence and apoptosis, thereby curtailing excessive tumor cell proliferation(Dou et al., 2017). Besides its activity subjected to be precisely regulated, Lv et al. discovered that cGAS transcription is epigenetically modulated by TET2, facilitating vascular normalization and antitumor immune responses in liver cancer(Lv et al., 2024b).

Paradoxically, cGAS activation can also facilitate tumorigenesis, progression, and metastasis. cGAMP, the product of cGAS, can diffuse to adjacent cells via gap junctions. In astrocytes, this influx of cGAMP elicits cGAS–STING signaling alongside the production of proinflammatory cytokines, fueling metastatic brain disease(Chen et al., 2016b). Tumor cells likewise exhibit formidable adaptive capacities to circumvent innate immune surveillance by releasing various immunosuppressive substances to reconfigure the tumor microenvironment and inhibit immune cell function(Caronni et al., 2018), thus impeding activation of cGAS–STING–IFN-I signaling. Recent work revealed that solid tumors bearing mutant IDH1 silence the cGAS gene through epigenetic modifications. As a result, inhibitors of mutant IDH1 reverse DNA methylation, restore cGAS expression, and reawaken antitumor immunity(Wu et al., 2024a).

### 6.4 Aging

Cells become senescent and transform into a SASP with age, with a loss of tissue and cellular homeostasis and accompanied by senescence-associated inflammation and impaired tissue function(Childs et al., 2017). The cGAS-STING-IFN-I pathway is activated in the retinas of aged mice and primary human fibroblasts from elderly donors while inhibiting mitophagy could potentially reduce this activation and alleviate the loss of neuronal function(Jiménez-Loygorri et al., 2024). Aging is also associated with the de-repression of endogenous retroviral elements(De Cecco et al., 2019; Liu et al., 2023c) and mitochondrial dysfunction(Guilbaud et al., 2024; Gulen et al., 2023; Victorelli et al., 2023). In senescent cells, cytoplasmic retrotransposons activate and initiate a cGAS-dependent IFN-I response. Therefore, lamivudine treatment, which inhibits the transcription of these retrotransposons, alleviates signs of senescence in naturally aging mice and progeria models(De Cecco et al., 2019; Liu et al., 2023c). Recently, the Ablasser team conducted a systematic study focusing on the brains of aging mice and found that in senescent microglia, mitochondria are damaged and release DNA into the cytoplasm, triggering cGAS activation, as well as neurodegeneration and cognitive decline(Gulen et al., 2023). During physiological aging, YAP/TAZ activity in stromal cells decreases, which drives senescence by activating cGAS-STING signaling, contributing to tissue aging. As a result, maintaining YAP function can rejuvenate old cells and resist the onset of aging-related features(Sladitschek-Martens et al., 2022). Additionally, aberrant cGAS-STING activation is observed in cells from patients with aging-related diseases like ataxia-telangiectasia, Hutchinson-Gilford progeria syndrome (HGPS), and Werner syndrome. These findings suggest that the cGAS-mediated STING signaling pathway could be a potential target for intervening in aging-related diseases.

## 7 Functions of cGAS beyond cGAS-STING signaling

### 7.1 Genomic stability regulation

Recent investigations have illuminated a critical role for cGAS in DNA damage repair processes, operating independently of STING signaling. Within the nuclear compartment, cGAS interacts with PARP1, disrupting the formation of the PARP1-Timeless complex and ultimately inhibiting homologous recombination (HR)(Liu et al., 2018a). Jiang et al. have also proposed that cGAS functions as a negative regulator of HR repair, promoting micronucleus formation and inducing cellular demise under conditions of genomic stress(Jiang et al., 2019). Nevertheless, the functional consequence of cGAS in DNA damage repair processes is not uniformly detrimental. Zhen et al. have reported that in DNA damage-induced senescent cells, nuclear cGAS can suppress LINE-1 (L1) retrotransposition through a CHK2-cGAS-TRIM41-ORF2p regulatory axis, thereby contributing to the maintenance of genomic stability(Zhen et al., 2023). Additionally, Li et al. found that cGAS binds CDK1, inhibiting RNF8 recruitment to chromosome ends and reducing end fusion at DNA double-strand break (DSB). This suppression of non-homologous end joining (NHEJ)-mediated DSB repair during mitosis promotes cellular senescence(Li et al., 2022a). Additionally, cGAS binds replication fork proteins, inhibiting their movement and reducing abnormal replication under DNA damage, thus promoting genomic stability(Chen et al., 2020).

### 7.2 Cell death

While MRE11 is well-characterized for its role in DNA damage sensing and repair, MRE11 can facilitate the release of cGAS sequestered on nucleosomes, thereby initiating an innate immune response. Intriguingly, upon interaction with cGAS, MRE11 triggers ZBP1-dependent necroptosis. Necroptosis, in this context, functions as a protective mechanism to eliminate severely damaged normal cells prior to malignant transformation(Beltrán-Visiedo et al., 2024; Cho et al., 2024). Furthermore, cGAS might also promote cell death during mitotic arrest(Zierhut et al., 2019). cGAS typically remains inactive due to its tight association with nucleosomes within the nucleus. However, under conditions of mitotic distortion, low levels of cGAS trigger transcription-independent cell apoptosis through IRF3.

### 7.3 Others: proliferation, micronucleus removal, and angiogenesis

Recent investigations have unveiled novel functions of cGAS beyond its canonical role in cytosolic dsDNA sensing and STING signaling. Huang et al. reported that in an inflammatory injury microenvironment, activated cGAS exerts an inhibitory effect on normal endothelial cell proliferation by suppressing YAP activation(Huang et al., 2020). Hu and colleagues discovered that cGAS, which highly expresses in intestinal stem cells, plays a crucial role in maintaining intestinal barrier integrity. Thereby, genetic ablation of cGAS in mice exacerbates chemically induced colitis and colitis-associated colorectal cancer (CAC)(Hu et al., 2021). Moreover, it is well-documented that cGAS can recognize micronuclei characterized by disrupted nuclear membranes in the cytoplasm, thereby activating STING and inflammatory signaling pathways. However, recent research has uncovered an additional function for cGAS as a receptor mediating the engulfment of micronuclei, thus contributing to the reduction of micronucleus burden(Chen et al., 2023). Adding to the functional versatility of cGAS, a recent study indicates that cGAS can initiate the formation of tertiary lymphoid structures (TLSs) in the lung, specialized structures that enhance humoral and antitumor immunity, albeit potentially contributing to the pathogenesis of autoimmunity in certain contexts(Zhao et al., 2024). Additionally, vascular endothelial growth factor A (VEGF-A)-induced nuclear translocation of cGAS initiates a signaling cascade involving miR-212-5p and ARPC3 to mediate angiogenic processes(Luo et al., 2023). Intriguingly, a recent investigation revealed that nuclear cGAS can directly activate innate immune responses in dendritic cells (DCs) and macrophages during viral infection within the nuclear compartment. Specifically, upon HIV-2 entry into the nucleus, the viral capsid sensor NONO forms a complex with nuclear cGAS, facilitating cGAS detection of viral DNA and subsequent STING activation(Lahaye et al., 2018).

**Fig. 4:**
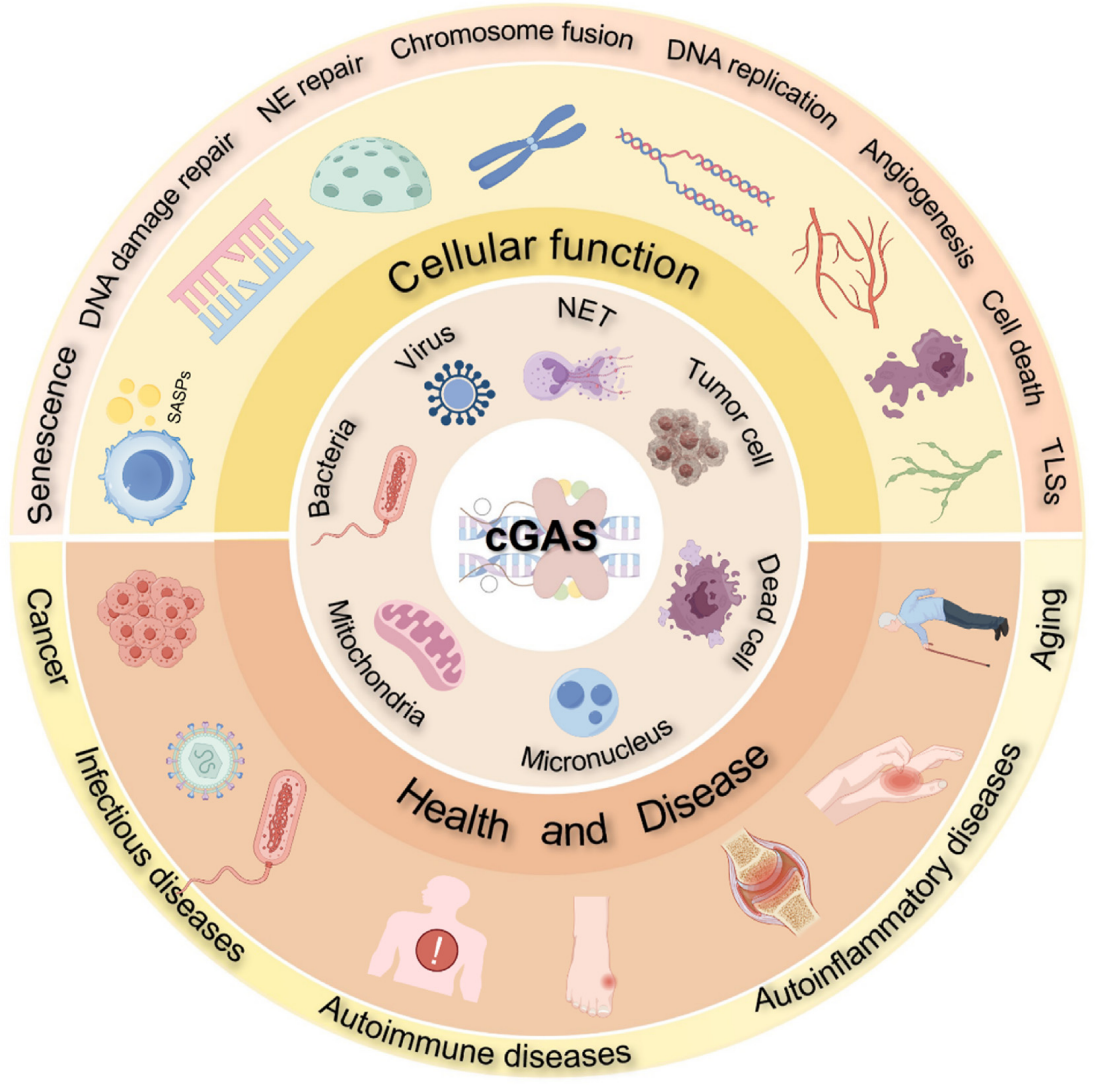
**Multifaceted Roles of cGAS in Cellular Physiology and Disease Pathogenesis.** This schematic illustration provides a comprehensive overview of the diverse functions of cGAS at both the cellular and systemic levels, highlighting its involvement in physiological processes and pathological conditions. **Abbreviation:** NET, neutrophil extracellular traps; NE, nuclear envelope; TLSs, tertiary lymphoid structures.

Therefore, despite recognizing cGAS as a critical innate immune sensor with increasingly diverse functions, our understanding of its functional repertoire remains limited (Fig. 4). Given the predominant nuclear localization of cGAS, it would be of considerable interest to investigate its interactions with checkpoint proteins across various cell cycle phases. Recent evidence indicates the presence of cGAS in diverse organelles, such as mitochondria and lysosomes, emphasizing the necessity for further investigation into its regulatory functions and underlying mechanisms within these subcellular compartments.

## 8 Pharmacological regulation of cGAS

The roles of cGAS-STING signaling in diverse cellular functions and diseases made it a prominent target for drug developing. Targeting cGAS may be more feasible for drug development than STING, as cGAS owns an active site that creates a suitable pocket for small molecule binding. Several cGAS antagonists have been discovered that inhibit cGAS activation by competitively binding dsDNA, including hydroxychloroquine (HCQ), quinacrine (QC), and ACMA(An et al., 2015), as well as actinomycin D(Bose et al., 2016), A151(Steinhagen et al., 2018), Suramin(Wang et al., 2018), ethidium bromide(Hu et al., 2022) and X6(An et al., 2018). An alternative strategy for developing cGAS inhibitors is to block its catalytic activity, such as RU.521(Lama et al., 2019; Vincent et al., 2017; Xu et al., 2020), which binds the enzyme active site and competes with ATP or GTP. Wang et al. developed a novel class of cGAS cyclic peptide inhibitors, such as XQ2B, which selectively target the cGAS-DNA interaction interface and block the formation of their aggregates(Wang et al., 2023b). It is worth noting that VENT-03, the first cGAS inhibitor to enter Phase I clinical trials, can significantly reduce inflammation in various organs(Mullard, 2023).

cGAS activators have also been granted significant attention, although their development has faced challenges. A 26-mer DNA sequence derived from the HIV-1 RNA genome is identified as a cGAS activator and is commercialized(Herzner et al., 2015). Moreover, Mn^2+^ has been found to activate cGAS and directly induce the production 2′3′-cGAMP(Wang et al., 2023a; Zhao et al., 2020b). Liu and colleagues discovered that Brivanib acts as a novel synergistic agent for cGAS, significantly enhancing the STING-TBK1-IFN-I response induced by cancer chemotherapy *in vitro* and *in vivo*(Liu et al., 2023a).

Comparative analyses of cGAS modification profiles in healthy versus diseased states can potentially delineate its specific roles in pathological processes. A refined understanding of these cGAS modification mechanisms could pave the way for the rational development of targeted small molecule modulators or biological therapeutics designed to either potentiate or inhibit cGAS function, aiming to treat specific immune-related diseases.

## 9 Perspectives

The established roles of cGAS in innate immunity, DNA damage repair, cell death, senescence, and angiogenesis collectively signify a substantial advancement in our understanding of this critical DNA sensor. Recent groundbreaking discoveries have challenged the initial characterization of cGAS as solely a cytoplasmic protein, revealing its presence and functional significance within diverse organelles. Intriguingly, a significant proportion of cGAS is thought to be associated with nucleosomes within the nucleus, where it orchestrates a few cellular functions. However, the regulatory factors governing the precise localization of cGAS across different subcellular compartments remain largely enigmatic. Moreover, the potential contribution of cytoplasmic cGAS to many pathological processes remains uncertain, especially in neurological and metabolic diseases. Given the attractive role of cGAS in senescence and organismal aging, future studies should leverage genetic manipulation and pharmacological inhibitors in established aging models to evaluate its functional contributions comprehensively. In addition, owing to the inherent complexity of tumor heterogeneity and etiological diversity, the regulatory functions of cGAS within the tumor microenvironment are multifaceted. Therefore, a detailed understanding of the activation status and regulatory mechanisms governing cGAS in specific tumor subtypes is pivotal for rationalizing targeted therapeutic interventions and effective combination treatment strategies. However, it is noteworthy that only a single cGAS inhibitor has progressed to clinical trials, highlighting the challenges of developing small molecule modulators that demonstrate robust efficacy in preclinical models. Finally, continued advancements in cutting-edge technologies, such as genome editing, single-cell genomics, cryo-ET, and advanced tissue imaging modalities, will undoubtedly empower systematic investigations into the context-dependent events governing cGAS function and disease pathogenesis.

## CRediT authorship contribution statement

**Yutong Liu:** Writing – original draft, Investigation, Formal analysis, Conceptualization. **Pinglong Xu:** Writing – review & editing, Validation, Supervision, Project administration, Funding acquisition, Formal analysis, Conceptualization.

## Declaration of competing interest

The authors declare the following financial interests/personal relationships which may be considered as potential competing interests: Pinglong Xu reports financial support was provided by 10.13039/501100004835Zhejiang University. Pinglong Xu reports a relationship with Zhejiang University that includes:. If there are other authors, they declare that they have no known competing financial interests or personal relationships that could have appeared to influence the work reported in this paper.
